# Anti-asthmatic fraction screening and mechanisms prediction of Schisandrae Sphenantherae Fructus based on a combined approach

**DOI:** 10.3389/fphar.2022.902324

**Published:** 2022-09-12

**Authors:** Fan Li, Bin Li, Jiushi Liu, Xueping Wei, Tingyan Qiang, Xinlu Mu, Yumeng Wang, Yaodong Qi, Bengang Zhang, Haitao Liu, Peigen Xiao

**Affiliations:** ^1^ Key Laboratory of Bioactive Substances and Resources Utilization of Chinese Herbal Medicine, Institute of Medicinal Plant Development, Chinese Academy of Medical Sciences and Peking Union Medical College, Beijing, China; ^2^ Engineering Research Center of Traditional Chinese Medicine Resource, Peking Union Medical College, Institute of Medicinal Plant Development, Ministry of Education, Chinese Academy of Medical Sciences, Beijing, China; ^3^ Animal Science and Technology College, Beijing University of Agriculture, Beijing, China

**Keywords:** active fraction screening, anti-asthma, molecular docking, network pharmacology, Schisandrae Sphenantherae Fructus, UPLC-Q/TOF-MS/MS

## Abstract

**Objective:** Schisandrae Sphenantherae Fructus (SSF) is a traditional Chinese medicine used to treat coughs and pulmonary inflammatory diseases. However, the pharmacodynamic material basis and mechanisms for SSF in asthma treatment remain unclear. This study aims to screen the anti-asthmatic fraction and verify the pharmacodynamic material basis, predict the potential mechanism, and verify the interaction ability between compounds and core targets.

**Methods:** First, three fractions from SSF were compared in terms of composition, comparison, and anti-asthmatic effects. Then, the ultra-performance liquid chromatography-quadrupole/time-of-flight-mass spectrometry/mass spectrometry (UPLC-Q/TOF-MS/MS) strategy was used to identify the compounds from the active fraction, and the anti-asthmatic efficacy of the active fraction was further studied by the ovalbumin (OVA)-induced asthma murine model. Finally, network pharmacology and molecular methods were used to study the relationships between active compounds, core targets, and key pathways of PEF in asthma treatments.

**Results:** The petroleum ether fraction (PEF) of SSF showed better effects and could significantly diminish lung inflammation and mitigate the level of serum immunoglobulin E (IgE), interleukin (IL)-4, IL-5, IL-6, IL-13, and IL-17 in mice. A total of 26 compounds from the PEF were identified, among which the main compounds are lignans and triterpenes. Moreover, 21 active compounds, 129 overlap-ping targets, and 10 pathways were screened by network pharmacology tools. The top five core targets may play a great role in asthma treatment. Gene Ontology (GO) analysis and Kyoto Encyclopedia of Genes and Genomes (KEGG) pathway analysis suggested that the PEF can treat asthma by acting on multiple asthma pathological processes, including the IL-17 signaling pathway, T helper (Th) 17 cell differentiation, and the calcium signaling pathway. Molecular docking was performed to evaluate the interactions of the protein–ligand binding, and most docked complexes had a good binding ability.

**Conclusion:** The present results might contribute to exploring the active compounds with anti-asthmatic activity.

## 1 Introduction

Asthma is a chronic disease that affects all age groups, and the prevalence of asthma has been increasing annually (Dharmage et al., 2019). It has become a global health issue and a tremendous burden for the healthcare system and patients (Soriano et al., 2017). Although inhaled corticosteroids and β-adrenoceptor agonists are mainstay treatments, they are prone to adverse reactions and drug resistance (Waldeck, 2002; Horne, 2006). Therefore, there has been an increasing focus on safe complementary and alternative medicine (CAM) treatments, among which traditional Chinese medicine (TCM) is considered to be an important part (Ernst, 2000). TCM is commonly used for asthma treatment and has attracted increasing attention due to its good clinical effects, few adverse reactions, and high safety (Bielory, 2004). Thus, there is much interest in identifying more effective drugs among TCM for asthma treatment.

Schisandrae Sphenantherae Fructus (SSF), sourced from *Schisandra sphenanthera* Rehd. et Wils. (Schisandraceae), is commonly used for treatment of coughs and pulmonary and inflammatory diseases and has been officially listed in the Chinese Pharmacopoeia (The State Pharmacopoeia Commission of China, 2020). According to classical books of ancient Chinese medicine, SSF is good at treating coughs and asthma (Li et al., 2019). However, evidence-based research into efficacy, pharmacodynamic material basis, and potential mechanisms of SSF against asthma remains in its infancy. In addition, it is difficult to uncover the material basis and underlying mechanisms of SSF against asthma solely using traditional pharmacological methods. Therefore, a combined approach including rapid identification of compounds, pharmacological experiments, and computer analysis was established in this article.

In this study, three fractions of SSF, namely, the petroleum ether fraction (PEF), ethyl acetate fraction (EAC), and a higher polar fraction (HPF) were compared in terms of UPLC chromatography and anti-asthmatic effects in an ovalbumin (OVA)-induced allergic asthma model. The pharmacodynamic material basis of the active fraction was studied by the ultra-performance liquid chromatography-quadrupole/time-of-flight-mass spectrometry/mass spectrometry (UPLC-Q/TOF-MS/MS) system, and the anti-asthmatic efficacy of the active fraction was further studied. Then, an *in silico* network pharmacology method was applied to predict the active compounds, core targets, and potential pathway of the active fraction against asthma. Finally, molecular docking was applied to verify the results of network analysis.

## 2 Materials and methods

### 2.1 Materials

SSF samples were collected from Hanzhong city (33°35′N, 106°49′E), Shaanxi province of China. The authentication of the voucher specimens was identified by Yaodong Qi and Xueping Wei, researchers of the Institute of Medicinal Plant Development (IMPLAD), Beijing, China. The specimens were deposited in the Medical Plant Resource Research Center of IMPLAD.

### 2.2 Pharmacological experiments

#### 2.2.1 Animals

Male C57BL/6 mice (20–22 g) were purchased from SPF Biotechnology Co., Ltd. (license number: SCXK [Beijing] 2019-0010) (Beijing, China). All animal experiments were approved by the Institutional Animal Care and Use Committee of IMPLAD (ethics approval number: SLXD-20210115027) and were carried out in agreement with the Guide for the Care and Use of Laboratory Animals (NIH publication #85–23, revised in 1985). All mice were housed under a 12-h light–dark cycle in a temperature- and humidity-controlled room with free access to tap water and irradiated food.

#### 2.2.2 Mouse grouping and model establishment

In the animal experiment of active fraction screening of SSF, mice were randomly divided into the following seven groups (*n* = 6). A control group (CON) was treated with vehicle (0.9% physiological saline) every other day from days 27–48 and saline sensitization/challenge. A model group (MOD) was treated with vehicle (0.9% physiological saline) every other day from days 27–48 and OVA sensitization/challenge. A positive control group (POS) was treated with 1 mg/kg dexamethasone every other day from days 27–48 and OVA sensitization/challenge. Also, four administration groups (SSF, PEF, EAC, and HPF) were treated with 200 mg/kg SSF, PEF, EAC, and HPF every other day from days 27–48 and OVA sensitization/challenge.

In the anti-asthmatic animal experiment of the PEF, mice were randomly divided into the following six groups (*n* = 6). The control group (CON) was treated with vehicle (0.9% physiological saline) every other day from days 27–48 and saline sensitization/challenge. The model group (MOD) was treated with vehicle (0.9% physiological saline) every other day from days 27–48 and OVA sensitization/challenge. The positive control group (POS) was treated with 1 mg/kg dexamethasone every other day from days 27–48 and OVA sensitization/challenge. Also, three PEF groups (low, medium, and high) were treated with 100, 200, and 400 mg/kg PEF every other day from days 27–48 and OVA sensitization/challenge, respectively.

The asthma model was established referring to the previous description with minor changes (Ma et al., 2021). The experimental mice were sensitized by intraperitoneal injection of 50 μg OVA (Sigma, St. Louis, MO, United States) with 100 μl alum adjuvant (Thermo Fisher Scientific, Foster City, CA, United States) on days 0, 7, and 14 and then challenged with 5% OVA for 30 min from days 21–23. From days 27–45, an inhalational challenge with 5% OVA was performed every other day. From days 46–48, the mice in the asthma group were challenged with endotracheal instillation of OVA (60 μg). The control mice underwent an identical schedule for sham-inducing conditions by saline. The mice were given intragastric administration every other day from the 27th day onward, 30 min before the OVA challenge. The dose of administration groups was determined based on our previous research on the anti-asthmatic activity of a subfraction from *Schisandra chinensis* fruit extract, literature reference, and pre-experiment (Li et al., 2022). At last, the mice were killed on day 49 after overnight fasting, mouse lung tissues were collected for histological examination, and blood samples were collected for Elisa examination.

#### 2.2.3 Pulmonary histopathology and Elisa

The left lower lobe of the lungs was removed, fixed in 10% formalin, and sectioned at 4 μm for hematoxylin–eosin (HE) staining to assess inflammation of the lungs. Pathological assessment of lung inflammation was graded blindly on a scale of 0–4 (least to most severe) based on an assessment of the quantity and quality of peribranchial infiltration of inflammatory cells, perivascular infiltration of inflammatory cells, and interstitial inflammation. The degree of inflammation and lesions of each slide was graded as follows: 0, no inflammation or lesion; 1, minimal; 2, mild; 3, moderate; and 4, severe. To measure the inflammatory responses, sera from the mice were collected. The levels of total IgE and inflammatory cytokines in serum were determined using the Elisa kits according to the manufacturer’s instructions.

### 2.3 UPLC-Q/TOF-MS/MS analysis

#### 2.3.1 UPLC-Q/TOF-MS/MS methods

UPLC separation was achieved on a Waters ACQUITY I-Class system (Waters Corporation, Milford, MA, United States) using a Waters ACQUITY BEH C_18_ column (2.1 × 100 mm, 1.7 μm, MA, United States). The column and autosampler were maintained at 30 and 10°C, respectively. The flow rate was set at 0.3 ml/min, and the injection volume was 1 μl. The mobile phase consisted of water (A) and acetonitrile (B), and the gradient conditions were as follows: 0–4 min, 42–50% B; 4–6 min, 50–48% B; 6–12 min, 48% B; 12–16 min, 48–62% B; 16–21 min, 62% B; 21–22 min, 62–85% B; 22–29 min, 85–95% B; 29–34 min, 95% B. The online UV spectra were recorded in the range of 200–400 nm.

Mass spectrometry was operated on the Waters Xevo G2-XS Q/TOF mass spectrometer (Waters Corporation, Milford, MA, USA) equipped with an electrospray ionization (ESI) source controlled by MassLynx 4.2 software (Waters, Corporation, Milford, MA, United States). A full scan was run in positive and negative modes, with a mass range from *m/z* 100–1,200 Da and with a 1 s scan time. Nitrogen was used as a nebulizer and auxiliary gas. In positive ion mode, the following parameters were found: capillary voltage, 3 kV; sampling cone voltage, 20 V; source temperature, 100°C; desolvation temperature, 350°C; cone gas flow, 50 l/hr; desolvation gas flow, 600 l/hr, and collision energy, 6 eV. Leucine enkephalin was used as a lock mass with a reference mass value at *m/z* 556.2771. MS/MS fragment information was obtained using a collision energy ramp from 10 to 45 eV. In the negative ion mode, the following parameters were found: capillary voltage, 3 kV; sampling cone voltage, 40 V; source temperature, 100°C; desolvation temperature, 350°C; cone gas flow, 50 l/hr; desolvation gas flow, 600 l/hr, and collision energy, 32 eV. The lock mass solution was Leucine enkephalin with a reference mass value at *m/z* 554.2615.

#### 2.3.2 Data processing

The raw data were imported into Progenesis QI (Waters Corporation, Milford, MA, United States). After automatic processing, alignment, peak picking, normalization, and deconvolution, the ingredients of the PEF were tentatively identified on the grounds of the in-house database we constructed before (Supplementary Table S1). Then, the ion information about the compounds was obtained by Masslynx software (Waters Corporation, Milford, MA, United States). Compounds were then identified by comparing MS spectra with standards. Moreover, the fragmentation rules about characteristic fragments and neutral fragments of these compounds were compared with those in the related literature and reference experiment results to further confirm the ingredients.

### 2.4 Network pharmacology analysis

#### 2.4.1 Active ingredients and putative targets collection

Compounds from the active fraction were identified by the UPLC-Q/TOF-MS/MS method. The SMILES (Simplified Molecular Input Line Entry System) was entered in the SwissADME (http://www.swissadme.ch, accessed on 11 December 2021) to assess the properties of pharmacokinetics and drug-likeness of compounds. Then, the SwissTargetPrediction (http://www.swisstargetprediction.ch/index.php, accessed on 11 December 2021) was employed to predict the putative targets of active compounds (Gfeller et al., 2014).

#### 2.4.2 Asthma-related targets collection

Asthma-related targets were searched from the following databases: Therapeutic Target Database (http://db.idrblab.net/ttd/), OMIM (https://www.omim.org/), DrugBank (http://www.drugbank.ca/), and GeneCards (https://www.genecards.org/) (all accessed on 12 December 2021) (Chen, 2002; Hamosh, 2004; Safran et al., 2010; Wishart et al., 2018). In addition, the GEO database was applied to retrieve asthma-related targets by using “asthma” as the keyword (https://www.ncbi.nlm.nih.gov/geo/) (accessed on 12 December 2021). All asthma-related targets were carried out by jvenn online tools (http://jvenn.toulouse.inra.fr/app/example.html, accessed on 12 December 2021) (Bardou et al., 2014).

#### 2.4.3 Protein–protein interaction (PPI) analysis

The overlapping targets between the active fraction and asthma were carried out by jvenn online tools (accessed on 12 December 2021). The STRING database (https://string-db.org, accessed on 12 December 2021) was then applied for PPI analysis (Mering, 2003). The PPI network of the top five genes by degree ranking was visualized by the cytoHubba plugin (accessed on 12 December 2021) (Shannon et al., 2003; Chin et al., 2014).

#### 2.4.4 Enrichment analysis, inference of upstream pathway activity, and network construction

The Gene Ontology (GO) function enrichment and Kyoto Encyclopedia of Genes and Genomes (KEGG) pathway analysis were constructed using the WebGestalt database (http://www.webgestalt.org, accessed on 13 December 2021) (Wang et al., 2017). For GO enrichment, biological processes (BP), cellular components (CC), and molecular functions (MF) were analyzed. The upstream activation or inhibition pathway of the overlapping targets was predicted with the SPEED2 online tool (https://speed2.sys-bio.net, accessed on 13 December 2021) (Rydenfelt et al., 2020). Then, Cytoscape 3.8.0 was applied to construct the network of the PEF–component–target pathway. In the network, the edges represent interactions between the nodes, and the nodes represent the PEF, active compounds, related targets, or signaling pathways.

### 2.5 Molecular docking

Finally, molecular docking was applied to visualize the patterns of interactions between the compounds and the top five targets. The compounds which have relationships with the top five targets were selected to conduct the molecular docking analysis and their structures were retrieved from the PubChem database (accessed on 11 December 2021). Then, PDB formats were obtained by Open Babel 3.1.1, and energy minimization was employed by an MMFF94 force field. AutoDockTool 4.2.6 was used to convert the PDBQT formats (Morris et al., 2009). The structures of the core targets were obtained from the RCSB Protein Data Bank (PDB database, http://www.rcsb.org/) (accessed on 13 December 2021) (Sussman et al., 1998). AutoDockTool 4.2.6 was applied to delete non-standard amino acids and add hydrogens and partial charges for targets, and then the structures of targets were converted to the PDBQT formats.

The molecular docking processes were carried out using AutoDock vina 1.2., and each docking task was repeated nine times with the exhaustiveness parameter of 16 and energy thresholds of ±2 kcal/mol (Trott and Olson, 2009). The lower the binding affinity is, the more stable the ligand–receptor conformation is. The docking pattern was applied to the rigid receptor–flexible ligand docking method, and the energy grid box was set at the geometrical center of the reference ligand within 15 Å.

### 2.6 Statistical analysis

Data were expressed as mean (SD). Statistical analysis was performed using Prism 8.4.0. (GraphPad, San Diego, CA, United States). Statistical comparisons were carried out based on was assessed by one-way analysis of variance (ANOVA). *p* < 0.01 indicated significant differences.

## 3 Results

### 3.1 UPLC comparison of SSF, PEF, EAC, and HPF

**FIGURE 1:**
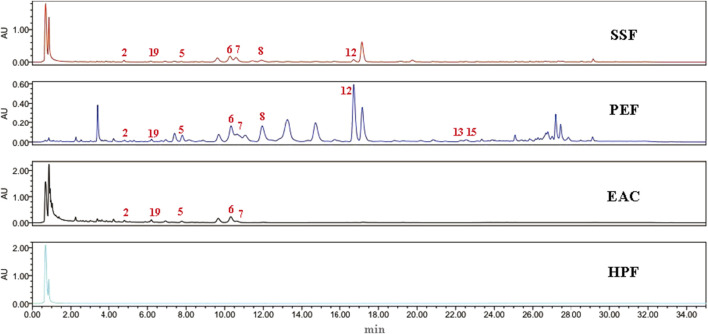
UPLC chromatography of SSF, PEF, EAC, and HPF. 2, gomisin J; 19, pregomisin; 5, gomisin G; 6, schisantherin A; 7, schisantherin B; 8, schisanhenol; 12, deoxyschizandrin; 13, benzoylgomisin O; and 15, angeloylgomisin O.

UPLC chromatograms of SSF, PEF, EAC, and HPF could be seen in Figure 1. Following a comparison of the retention time with standards, the nine peaks were identified to be gomisin G, gomisin J, pregomisin, schisanhenol, schisantherin A, schisantherin B, deoxyschizandrin, benzoylgomisin O, and angeloylgomisin O. Compared with other fractions, the PEF has more common compounds with SSF than other fractions.

### 3.2 Active fraction screening of SSF

**FIGURE 2:**
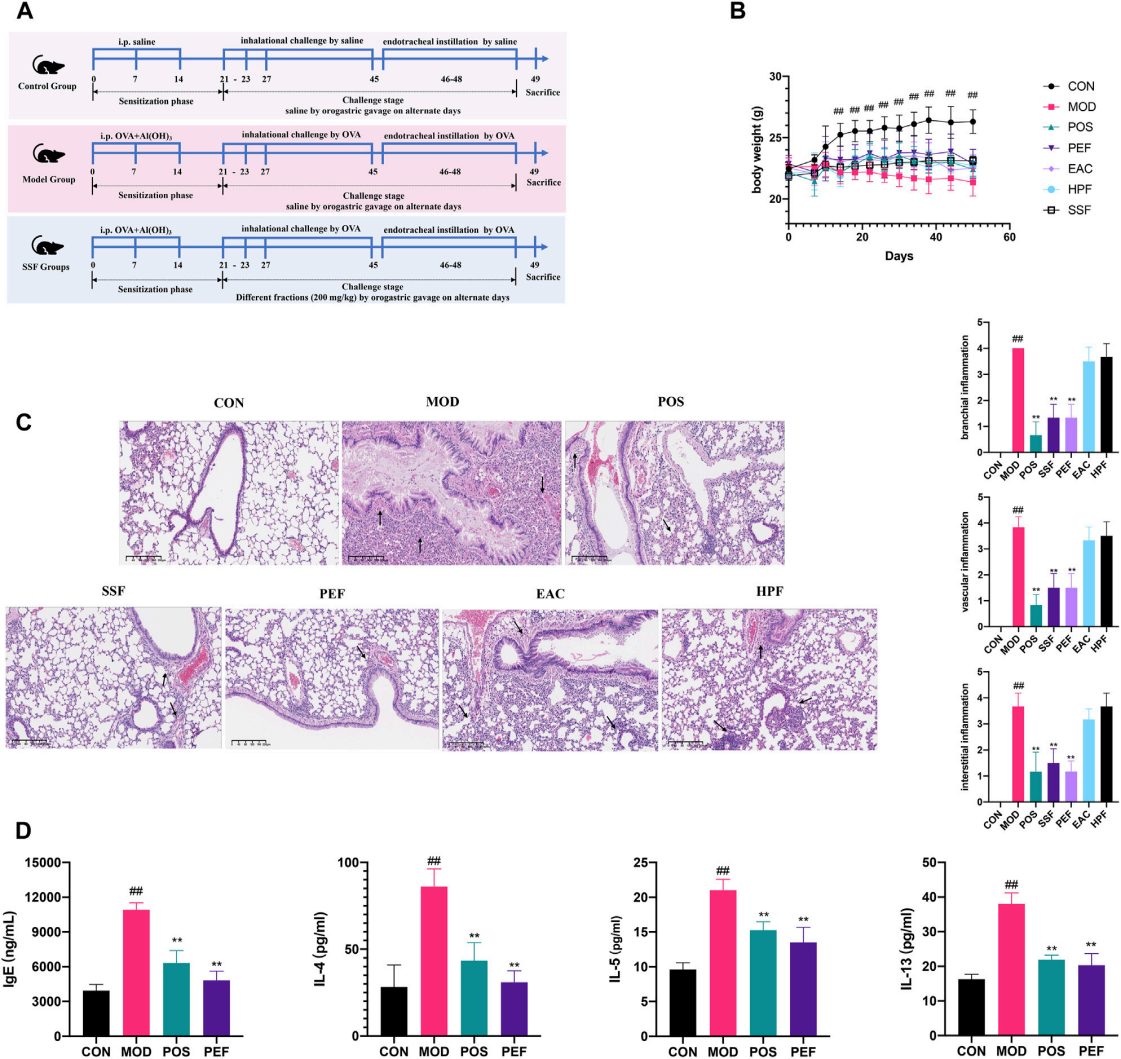
Active fraction screening of SSF. **(A)** Timeline of the animal experimental protocol. **(B)** Bodyweight records of the mice during the experiment (*n* = 6). **(C)** Pulmonary HE staining (× 10) and inflammation scores. **(D)** Expressions of inflammatory cytokines in serum. (##*p* < 0.01 v. s. CON group. **p < 0.01 v. s. MOD group. *p < 0.05 v. s. MOD group).

The anti-asthmatic effects of three fractions of SSF in an OVA-induced asthma model were evaluated (Figure 2A). The body weight of the control mice steadily increased during the experiment, and this trend experienced a significant increase after 14 days compared with that in the model mice (*p* < 0.01) (Figure 2B). However, the body weight of the mice in the model group decreased significantly, and the asthmatic mice exhibited behavioral troubles with irritability, agitation, shortness of breath, and scratching of the nose. Compared to other fractions of SSF, the PEF administration group showed milder asthma symptoms, and the morphological changes were not as severe as in other groups.

HE staining revealed that pulmonary structures were changed significantly in the model group, and the asthma model was successfully established. Compared to the control group, the airway injury was significant, peribranchial and perivascular inflammatory cells were infiltrated around the airway, and interstitial inflammation can be observed in the model mice (*p* < 0.01) (Figure 2C). Compared to other fractions of SSF, results of HE scoring showed that the mice in the PEF group showed less infiltration of inflammatory cells around the bronchioles, blood vessels, and alveoli (*p* < 0.01). Therefore, the staining results showed that the PEF is the most potent fraction. Serum IgE and interleukin (IL)-4, IL-5, and IL-13 were all elevated markedly in the model mice (*p* < 0.01), and these characteristics were substantially improved in the PEF group (*p* < 0.01) (Figure 2D). All the results indicated that the PEF is the active antiasthmatic fraction of SSF.

### 3.3 Identification of compounds from the PEF by UPLC-Q/TOF-MS/MS

**FIGURE 3:**
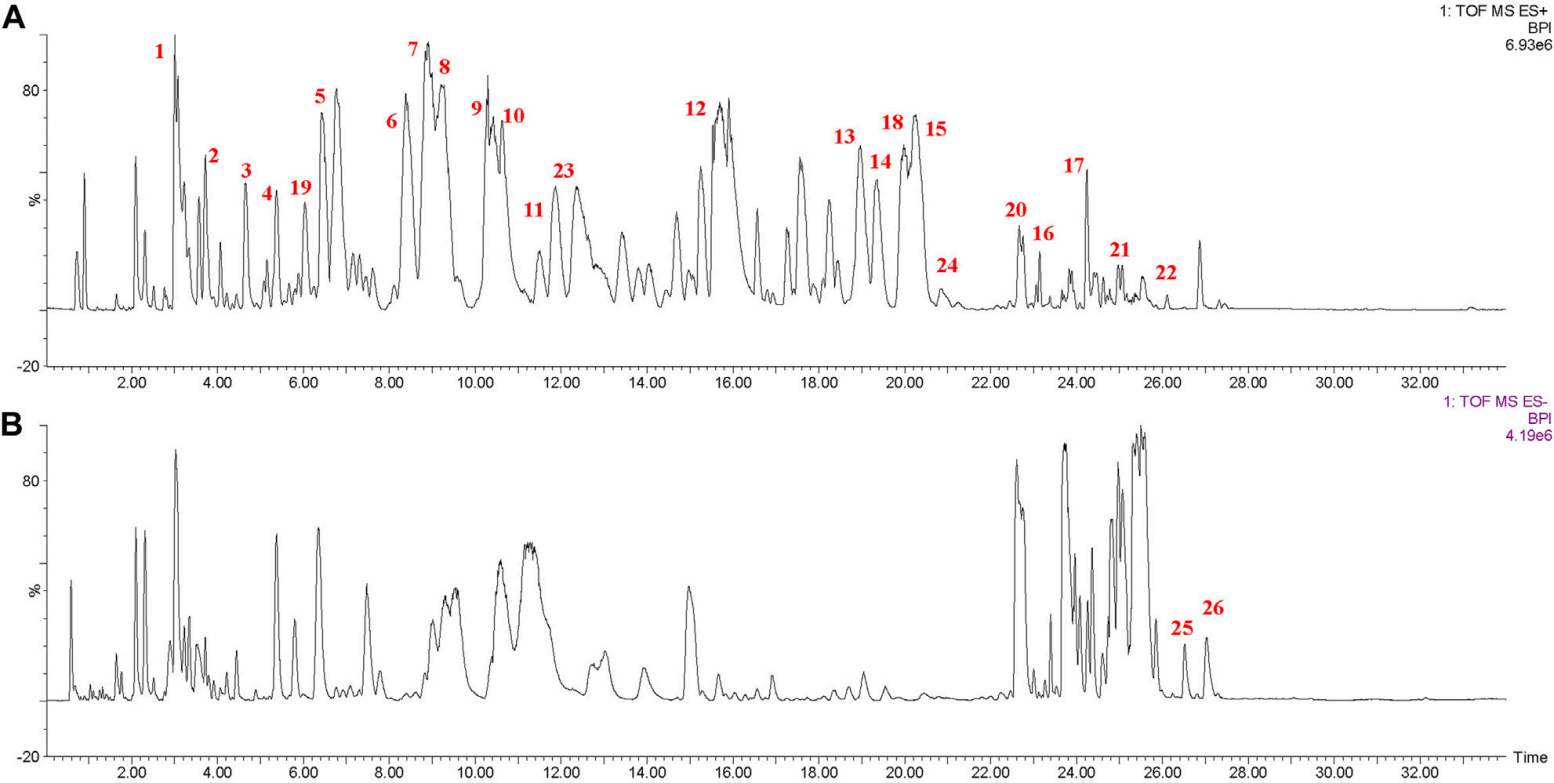
Base peak chromatogram of the PEF in positive **(A)** and negative **(B)** ion modes determined by the UPLC-Q/TOF-MS/MS method. 1, schisantherin E; 2, gomisin J; 3, schisandrin; 4, schisandrol B; 5, gomisin G; 6, schisantherin A; 7, schisantherin B; 8, schisanhenol; 9, gomisin F; 10, interiotherin A; 11, schisandrin C; 12, deoxyschizandrin; 13, benzoylgomisin O; 14, angeloylgomisin H; 15, angeloylgomisin O; 16, schisantherin D; 17, gomisin M2; 18, d-epigalbacine; 19, pregomisin; 20, schisphenthin A; 21, kadsuric acid; 22, kadsuric acid 3-methylester; 23, schinalactone A; 24, sphenadilactone E; 25, lancifodilactone B; and 26, lancifodilactone C.

Figure 3 shows the base peak chromatogram of the PEF in positive and negative ion modes. Following a comparison of the retention time and fragmentation regularity with standards, in-house database, and literature, 26 compounds, including 17 dibenzocyclooctadienes, one tetrahydrofuran, one dibenzylbutane, three lanostanes, one cycloartane, and three nortriterpenes, were identified by the UPLC-Q/TOF-MS/MS method. More detailed information on these identified compounds, including their retention time, adducts, confidence score, molecular formula, fragmentation score, mass error, and isotope similarity, is shown in Table 1.

**TABLE 1:** Identification of compounds from the PEF.

No.	RT^a^	Adduct	MF^b^	CS^c^	FS^d^	ME^e^	IS^f^	Compound	Ref
Dibenzocyclooctadienes									
1	3.2774	[M + NH_4_]^+^, [M + Na]^+^	C_30_H_34_O_9_	40.2	10.0	4.31	96.05	Schisantherin E	Huang et al. (2011)
2	4.2030	[M + H]^+^, [M + Na]^+^	C_22_H_28_O_6_	37.9	11.6	3.94	82.72	Gomisin J	Yang et al. (2017)
3	5.0652	[M + Na]^+^	C_24_H_32_O_7_	40.9	12.6	4.74	97.29	Schisandrin	Jiang et al. (2016)
									Yang et al. (2017)
4	5.7256	[M + H-H_2_O]^+^, [M + H]^+^	C_23_H_28_O_7_	50.5	61.4	4.50	96.37	Schisandrol B	Jiang et al. (2016)
									Yang et al. (2017)
5	6.9926	[M + H-H_2_O]^+^, [M + NH4]^+^, [M + Na]^+^	C_30_H_32_O_9_	41.4	13.6	−3.37	97.62	Gomisin G	Jiang et al. (2016)
									Yang et al. (2017)
6	8.7926	[M + NH_4_]^+^, [M + Na]^+^	C_30_H_32_O_9_	42.1	16.8	−3.37	97.62	Schisantherin A	Jiang et al. (2016)
									Yang et al. (2017)
7	9.1684	[M + H-H_2_O]^+^	C_28_H_34_O_9_	50.8	59.4	−2.75	97.91	Schisantherin B	Jiang et al. (2016)
									Yang et al. (2017)
8	9.6971	[M + NH_4_]^+^, [M + H]^+^, [M + Na]^+^	C_23_H_30_O_6_	44.2	24.2	2.03	69.48	Schisanhenol	Jiang et al. (2016)
									Yang et al. (2017)
9	10.1684	[M + Na]^+^	C_28_H_34_O_9_	51.0	60.2	−2.75	97.91	Gomisin F	Lee et al. (2013)
10	11.3831	[M + NH_4_]^+^	C_29_H_28_O_8_	37.5	6.18	−2.53	84.16	Interiotherin A	Lee et al. (2013)
11	11.7894	[M + H]^+^	C_22_H_24_O_6_	42.6	20.2	−2.59	95.97	Schisandrin C	Jiang et al. (2016)
									Yang et al. (2017)
12	15.8939	[M + H]^+^, [M + Na]^+^, [M + H + Na]^+^, [M + H-CH_3_-CH_3_OH]^+^, [M + NH_4_]^+^, [M + H-C_5_H_10_]^+^, [M + H-C_5_H_10_-OCH_3_]^+^, [M + H-C_5_H_10_-OCH_3_-CH_3_]^+^	C_24_H_32_O_6_	56.4	88.7	−1.88	85.43	Deoxyschizandrin	Jiang et al. (2016)
									Yang et al. (2017)
13	19.3984	[M + H]^+^	C_30_H_32_O_8_	48.2	47.9	−4.25	98.08	Benzoylgomisin O	Jiang et al. (2016)
									Yang et al. (2017)
14	19.8503	[M + Na]^+^	C_28_H_36_O_8_	43.9	29.2	0.95	91.35	Angeloylgomisin H	Lee et al. (2013)
15	20.9710	[M + NH_4_]^+^, [M + Na]^+^	C_28_H_34_O_8_	39.3	9.4	−0.51	97.26	Angeloylgomisin O	Lee et al. (2013)
16	23.6765	[M + Na]^+^	C_29_H_28_O_9_	51.1	60.0	0.66	96.08	Schisantherin D	Jiang et al. (2016)
									Yang et al. (2017)
17	24.5873	[M + H]^+^	C_22_H_26_O_6_	43.1	39.6	1.49	77.58	Gomisin M2	Lee et al. (2013)
Tetrahydrofurans									
18	20.1294	[M + H]^+^	C_20_H_20_O_5_	46.5	38.6	−0.54	94.33	d-Epigalbacine	Huang et al. (2011)
Dibenzylbutanes									
19	5.9736	[M + H]^+^, [M + Na]^+^	C_22_H_30_O_6_	38.5	14.4	−1.87	87.51	Pregomisin	Jiang et al. (2016)
									Yang et al. (2017)
Lanostanes									
20	22.8219	[M + NH_4_]^+^, [M + Na]^+^	C_30_H_46_O_5_	40.4	10.9	0.73	91.91	Schisphenthin A	Liu et al. (2017)
21	25.8671	[M + H]^+^	C_30_H_46_O_4_	42.4	19.5	−1.73	94.37	Kadsuric acid	Liu and Huang, (1984)
									Yang et al. (2017)
22	26.0657	[M-H-OCH_3_]^+^	C_31_H_48_O_4_	39.9	16.6	2.22	85.70	Kadsuric acid 3-methylester	Li, (2011)
									Yang et al. (2017)
Cycloartanes									
23	11.8773	[M + H]^+^	C_30_H_42_O_7_	35.3	10.9	−4.41	70.86	Schinalactone A	He et al. (2010)
Nortriterpenes									
24	21.5389	[M + H]^+^	C_29_H_34_O_10_	34.2	8.9	0.02	71.17	Sphenadilactone E	He et al. (2012)
25	26.1077	[M-H]^−^	C_29_H_34_O_11_	33.5	7.45	−2.83	71.00	Lancifodilactone B	Li, (2011)
									Yang et al. (2017)
26	27.1328	[M-H]^−^	C_29_H_36_O_10_	38.2	7.45	−0.99	92.05	Lancifodilactone C	Li, (2011)
									Yang et al. (2017)

a RT (retention time, min).

b MF (molecular formula).

c CS (confidence score).

d FS (fragmentation score).

e ME (mass error, ppm).

f IS (isotope similarity).

### 3.4 Pharmacological experiment of the PEF against asthma

**FIGURE 4:**
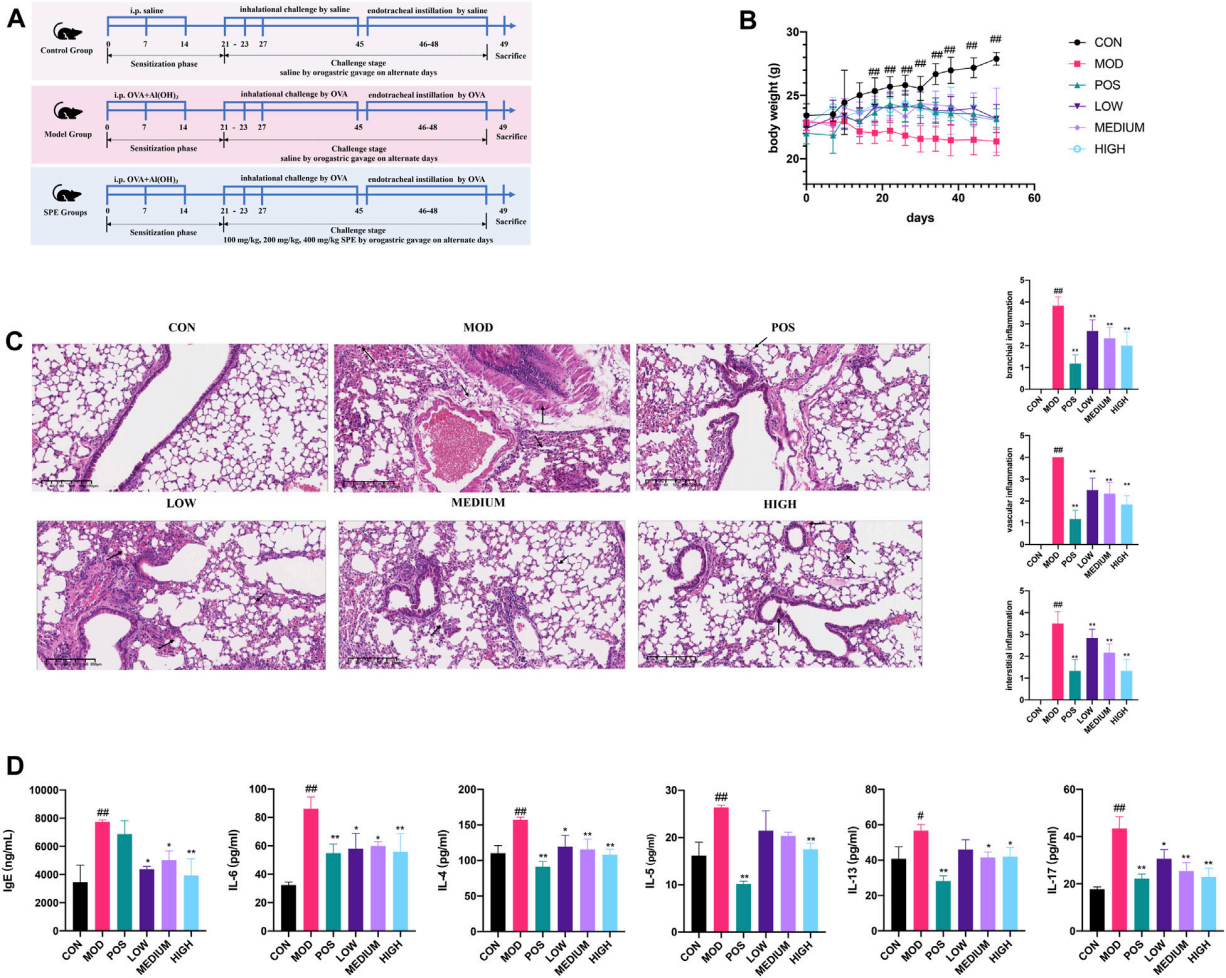
Validation of the PEF against asthma. **(A)** Timeline of the animal experimental protocol. **(B)** Body weight records of the mice during the experiment (n = 6). **(C)** Representative images of HE staining (× 10) and inflammation scores. **(D)** Expressions of inflammatory cytokines in serum. (##*p* < 0.01 v. s. CON group. **p < 0.01 v. s. MOD group. *p < 0.05 v. s. MOD group).

After screening the active fraction, the anti-asthmatic efficacy of the PEF was studied (Figure 4A). First, in the stages of nebulization and endotracheal instillation, the asthmatic mice showed significant weight loss, coughing, wheezing, sneezing, and mouth breathing, whereas the PEF groups showed milder asthma symptoms. Compared to the control mice, the model mice experienced a significant decrease during the later phase of sensitization (after 14 days) (*p* < 0.01) (Figure 4B). Then, the pulmonary histopathology and related scoring revealed that in the asthma mice, airway injury and infiltration of peribranchial and perivascular inflammatory cells can be observed obviously in the model mice. In the interstitial spaces of the model mouse lungs, HE staining revealed numerous infiltrations of inflammatory cells. HE staining and related scoring indicated that the anti-asthmatic model has been successfully constructed again (*p* < 0.01) (Figure 4C). In the PEF administration groups, a dose-dependent manner was observed based on the HE scores (*p* < 0.01). The high-dose group (400 mg/kg) showed the lowest peribranchial, perivascular, and interstitial inflammation scores, indicating a better effect of the anti-asthmatic effect. Then, the expressions of serum IgE, IL-4, IL-5, IL-6, IL-13, and IL-17 was significantly increased in the model group compared with those in the control group (*p* < 0.01). After the administration of the PEF, the expression of inflammation cytokines aforementioned was markedly decreased (*p* < 0.01). Among the three PEF groups, the mice in the 400 mg/kg PEF group demonstrated better therapeutic effects (Figure 4D). These findings imply that the PEF can significantly inhibit the pharmacological parameters of mice caused by OVA-induced asthma.

### 3.5 Network pharmacology analysis

#### 3.5.1 Active compounds and putative targets of the PEF

**FIGURE 5:**
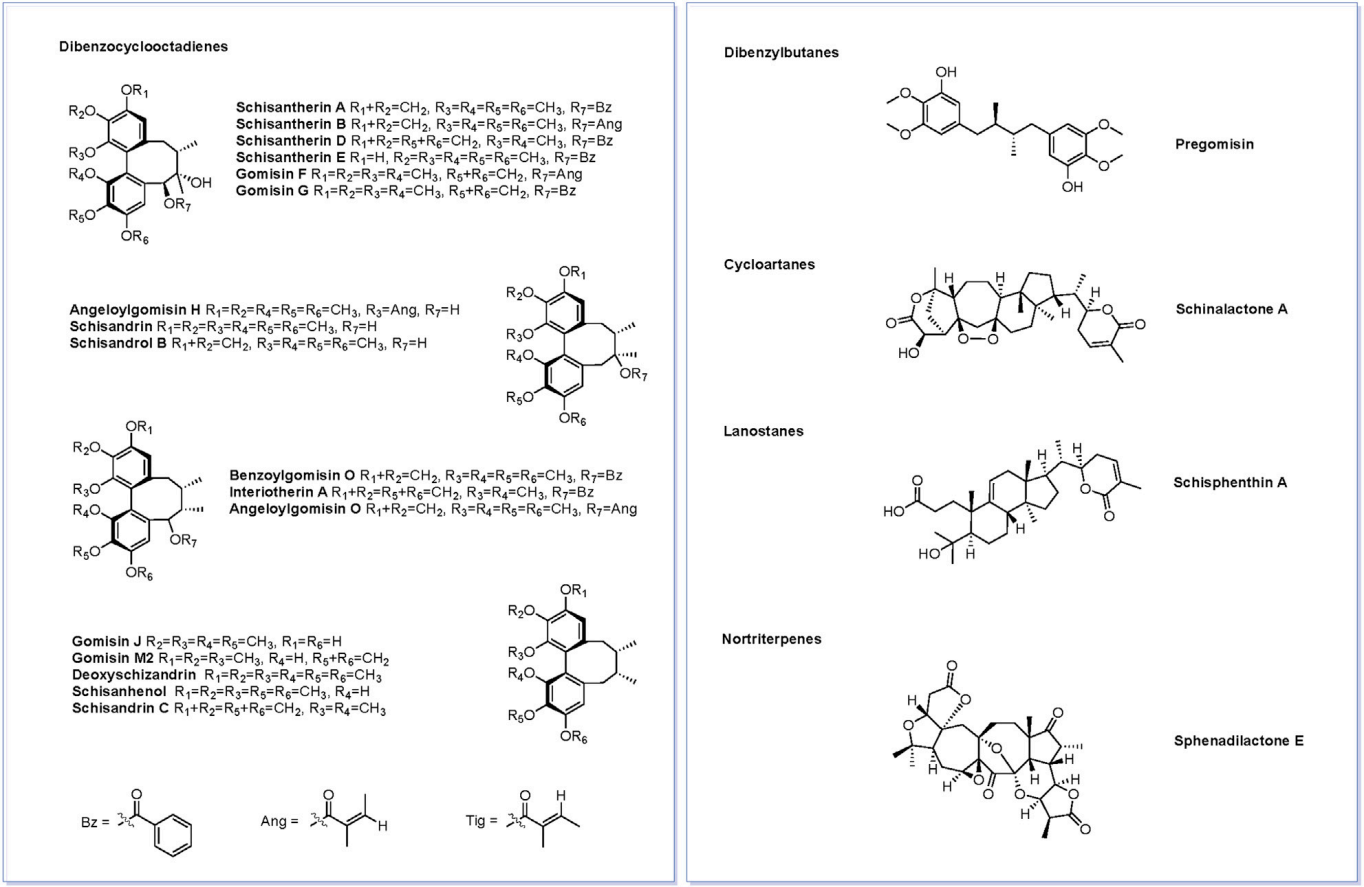
Structures of 21 active compounds screened based on the SwissADME.

Twenty-one compounds were regarded as bioactive ingredients by filtering with Lipinski’s rule and gastrointestinal absorption based on the SwissADME tool (Figure 5). According to Lipinski’s rule, the most common features of drug-like molecules include log*P* value (<5), molecular weight (<500), number of hydrogen bonds for the donor (≤5), and acceptor (≤10) (Daina et al., 2017). Duplicate targets were removed, and 549 putative targets were finally obtained by SwissTargetPrediction (Supplementary Table S2).

#### 3.5.2 Putative targets of asthma

**FIGURE 6:**
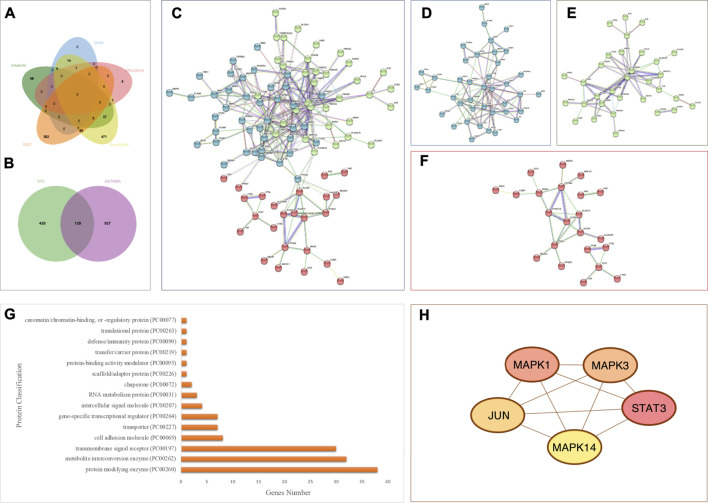
Analysis of asthma-related targets and overlapping targets between the PEF and asthma. **(A)** Venn diagram of asthma-related targets; **(B)** 129 overlapping targets. **(C)** Overall PPI analysis of 129 overlapping targets. **(D)** Cluster 1. **(E)** Cluster 2. **(F)** Cluster 3. **(G)** Protein classification by the Panther database. **(H)** Top five genes by degree ranking in the PPI using the cytohubba tool.

A total of 696 genes related to asthma were obtained from OMIM, DRUGBANK, Therapeutic Target Database, and GeneCards after removing the duplicate genes. On the other hand, four GEO datasets (GSE67472, GSE104468, GSE27876, and GSE64913) were retrieved from the GEO database using asthma as the keyword. The distribution of differentially expressed genes (DEGs) was illustrated by volcano plots and hierarchical clustering analysis (Supplementary Figure S1). We obtained 450 genes from these datasets (Supplementary Table S3). Finally, after removing the duplicate targets, 1,078 asthma-related targets were acquired (Figure 6A and Supplementary Table S4).

#### 3.5.3 Protein–protein interaction (PPI) analysis

A total of 129 overlapping targets between the PEF and asthma were obtained by jvenn online tools (Figure 6B and Supplementary Table S5), and the PPI analysis was carried out by the STRING platform (Figure 6C). Three clusters were obtained by the K-means clustering method. Cluster 1 (Figure 6D) has 44 genes (44 nodes and 96 edges, average node degree is 4.36, and average local clustering coefficient is 0.495). Cluster 2 (Figure 6E) has 40 genes (40 nodes and 60 edges, average node degree is 3, and average local clustering coefficient is 0.5). Cluster 3 (Figure 6F) has 44 genes (44 nodes and 29 edges, average node degree is 1.32, and average local clustering coefficient is 0.382). The Panther database was used for protein classification (Figure 6G). The top three protein classifications were the protein modifying enzyme (38 genes), metabolite interconversion enzyme (32 genes), and the transmembrane signal receptor (30 genes).

Then, the cytohubba tool was applied to obtain key genes. The top five genes by degree ranking in the PPI were visualized, namely, mitogen-activated protein kinase (MAPK) 14, JUN, signal transducer and activator of transcription (STAT)3, MAPK3, and MAPK1 (Figure 6H).

#### 3.5.4 Enrichment analysis and network construction

**FIGURE 7:**
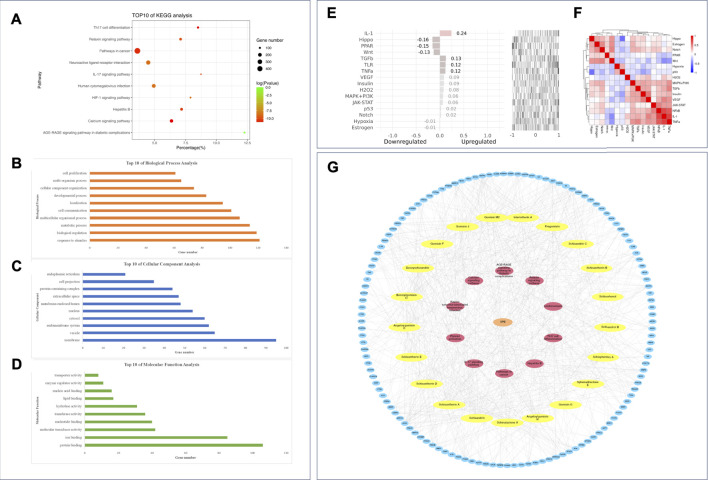
Enrichment analysis and network construction of the PEF against asthma. Top ten results of **(A)** KEGG analysis; **(B)** GO BP analysis; **(C)** GO CC analysis; **(D)** GO MF analysis. **(E)** Upstream pathway analysis of overlapping targets. **(F)** 16 pathways considered in upstream pathway analysis. **(G)** PEF–compound–target–pathway network.

The Gene Ontology annotation analysis and KEGG pathway analysis were carried out by webgestalt. The top ten pathways ranked by FDR were as follows: the AGE-RAGE signaling pathway in diabetic complications, the calcium signaling pathway, Th17 cell differentiation, pathways in cancer, hepatitis B, the IL-17 signaling pathway, the relaxin signaling pathway, the HIF-1 signaling pathway, neuroactive ligand–receptor interaction, and human cytomegalovirus infection (Figure 7A).

The top ten GO BPs were as follows: response to stimulus, biological regulation, metabolic process, multicellular organismal process, cell communication, localization, developmental process, cellular component organization, multi-organism process, and cell proliferation (Figure 7B). The top ten GO CCs were as follows: membrane, vesicle, endomembrane system, cytosol, nucleus, membrane-enclosed lumen, extracellular space, protein-containing complex, cell projection, and endoplasmic reticulum (Figure 7C). Also, the top ten GO MFs were as follows: protein binding, ion binding, molecular transducer activity, nucleotide binding, transferase activity, hydrolase activity, lipid binding, nucleic acid binding, enzyme regulator activity, and transporter activity (Figure 7D).

The SPEED2 tool was applied to explore the upstream pathway of the overlapping targets. IL-1, transforming growth factor β (TGFβ), toll-like receptor (TLR), tumor necrosis factor-α (TNF-α), vascular endothelial growth factor (VEGF), insulin, H_2_O_2_, janus kinase-signal transducer and activator of transcription proteins (JAK-STAT), mitogen-activated protein kinase-phosphoinositide-3-kinase (MAPK-PI3K), p53, and Notch were inferred upregulated with overlapping targets, and peroxisome proliferator-activated receptors (PPAR), Hippo, Wnt, hypoxia, and estrogen pathways were inferred downregulated upstream (Figures 7E and F). Based on the 21 active compounds, top five targets, and top ten pathways, a PEF–compound–target–pathway network was constructed using Cytoscape 3.8.0. (Figure 7G).

### 3.6 Molecular docking analysis

**FIGURE 8:**
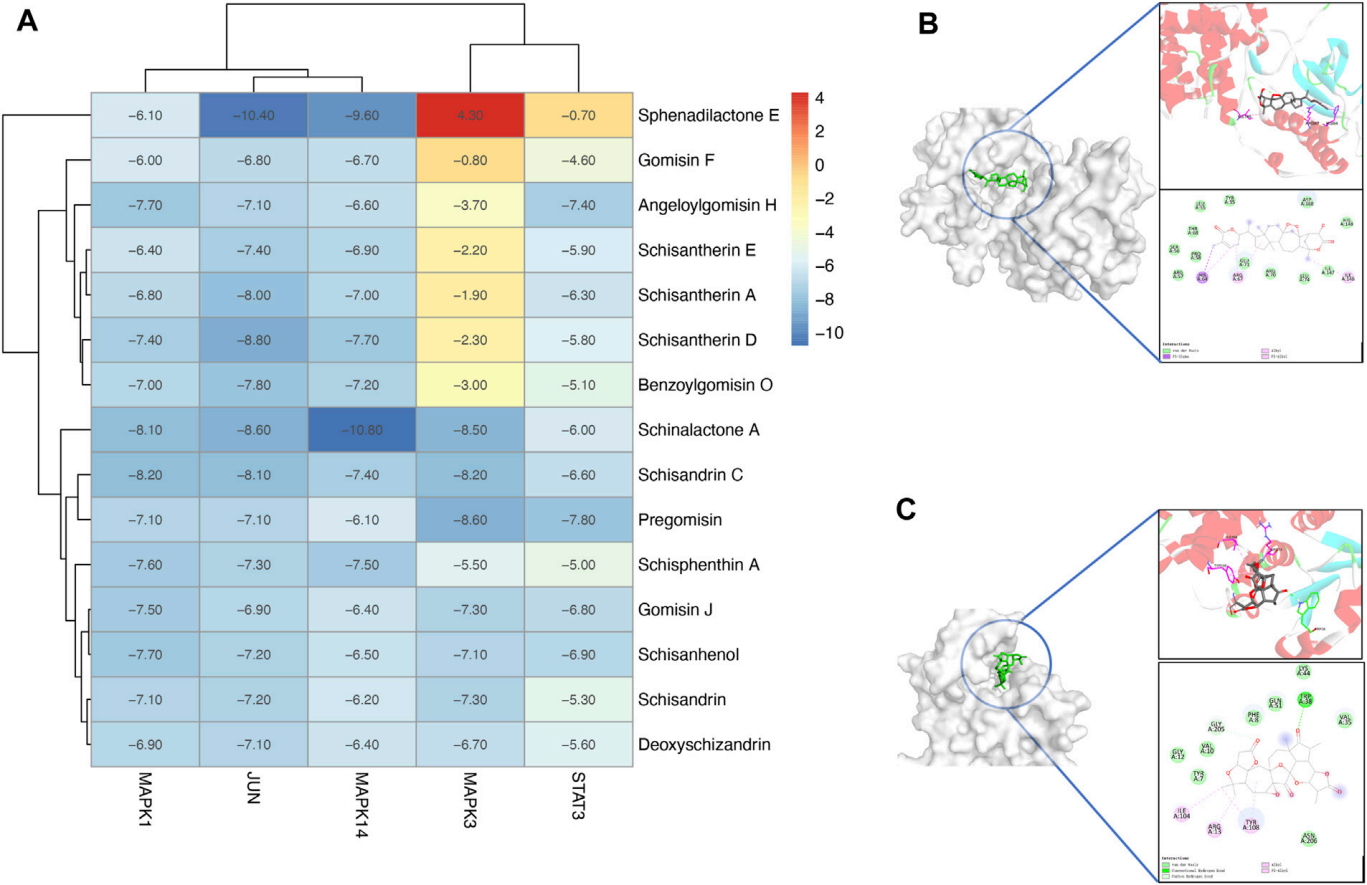
Molecular docking analysis of the PEF against asthma. **(A)** Heatmap of molecular docking analysis. **(B)** MAPK14 (PDB ID: 5WJJ)-Schinalactone A (−10.8 kcal/mol). **(C)** JUN (PDB ID: 5J41)-Sphenadilactone E (−10.4 kcal/mol).

An *in silico* molecular docking approach was performed to test the affinity between the compounds and the top five targets, namely, MAPK14 (PDB ID: 5ETF); JUN (PDB ID: 5J41); STAT3 (PDB ID: 6SM8); MAPK3 (PDB ID: 4QTB); and MAPK1 (PDB ID: 4ZZN). A total of 15 compounds that have relationships with the top five targets were selected for molecular docking analysis. In total, 75 docking simulations were executed. The RMSD values were stable, indicating stable interaction and accurate results. Also, according to the binding affinity scores, most complexes show negative results, indicating good interactions between the ligands and the receptors. Unexpectedly, lack of good interaction between MAPK3 (PDB ID: 4QTB) and sphenadilactone E was observed, and the docking result was a positive affinity value (Figure 8A).

Several ligand–target complexes have shown relatively high affinities among all results. For example, it was observed that schinalactone A docked on the MAPK14 (PDB ID: 5WJJ) and exhibited the strongest binding energy (−10.8 kcal/mol) (Figure 8B). Schinalactone A was found to interact with MAPK14 (PDB ID: 5WJJ) amino acids HIS64, ARG67, and ILE146 *via* π–alkyl interactions, and all of these interactions enhance a ligand-bound closed conformation. The ligand is bound to a narrow cleft pocket on the surface of the protein, which conforms to the shape structure of the ligand itself, reflecting a good shape match between the molecule and the protein. We also found a high affinity between JUN (PDB ID: 5J41) and sphenadilactone E (-10.4 kcal/mol) (Figure 8C). The results revealed that sphenadilactone E was bound in the cavity of JUN (PDB ID: 5J41), and JUN (PDB ID: 5J41) is shaped to dock into the structural features of their receptors, reflecting the good match between sphenadilactone E and JUN (PDB ID: 5J41). The ligand is bound at the cleft region of the target, and TRP38 formed hydrogen bond interactions with sphenadilactone E, while ILE104, ARG13, and TYR108 formed π–alkyl interactions with the ligand.

## 4 Discussion

In this study, we investigated the pharmacodynamic material basis and predicted the mechanisms of the active fraction from SSF in asthma treatment. The PEF may be the most potent anti-asthmatic fraction of SSF. A total of 26 compounds of the PEF were identified through the UPLC-Q/TOF-MS/MS technology, among which 21 components may be the active ingredients of asthma. A total of 129 overlapping targets between the PEF and asthma were obtained by jvenn online tools, among which five targets might play important roles: MAPK14, JUN, STAT3, MAPK3, and MAPK1. Then, the enrichment analysis revealed that the PEF might reduce inflammation by modulating Th2 cells, IL-17 signaling pathway, Th17 cell differentiation, and the calcium signaling pathway.

In the experiment of active fraction screening of SSF, a UPLC chromatography strategy and an OVA-induced allergic asthma murine model were constructed. The UPLC chromatograms showed that the PEF has more common compounds with SSF than other fractions. The OVA allergen-caused asthma is a typical airway inflammatory disease, and intraperitoneal sensitization with OVA/alum adjuvant is frequently used in experimental allergic asthma (Song et al., 2019). Pharmacodynamic experiments showed that the PEF mice showed less infiltration of inflammatory cells around the bronchioles, blood vessels, and alveoli. Our results revealed that the PEF had better anti-asthmatic activity.

To elucidate the material basis of the PEF, the plant metabolomics strategy was further employed for chemical composition characterization by the UPLC-Q/TOF-MS/MS technique. Abundant mass spectrometry information was obtained by scanning in the positive and negative ion modes, and the identification of chemical constituents was performed based on Progenesis QI software, standard substances, and related literature. Lignans, especially dibenzocyclooctadienes, are emerging as one of the primary phytochemicals in the PEF. Schisandrol B could inhibit the level of pro-inflammatory factors in an OVA-induced asthma murine model by inhibiting the nuclear factor-kappa B (NF-κB) pathway (Wu et al., 2018). Schisantherin A decreased the expression of proinflammatory cytokines in the bronchoalveolar lavage fluid (BALF) and blocked the lipopolysaccharide-induced acute respiratory distress syndrome by regulating the NF-κB and MAPK pathways (Zhou et al., 2014). Gomisin C limited the respiratory burst of rat neutrophils by inhabitation of nicotinamide adenine dinucleotide phosphate (NADPH) oxidase and cytosolic Ca^2+^ (Wang et al., 1994).

Then, in the experiment of PEF against asthma, our findings showed that the expression of IgE, IL-4, IL-5, IL-6, IL-13, and IL-17 were significantly higher in the model group. The oral administration of the PEF can reduce the production of the cytokines aforementioned. IgE is considered to be the first line of defense against pathogens and plays a significant role in the occurrence and development of asthma (Gould and Sutton, 2008). During allergic reactions, IgE sensitizes mast cells by binding the high-affinity IgE receptors on the surface of mast cells and antigen-presenting cells, causing the cells to degranulate and release inflammatory mediators. The presence of serum IgE is the prototypical hallmark of adaptive Th2 immunity, driven by IL-4-induced class switching of the immunoglobulins synthesized by B cells (Lambrecht and Hammad, 2015). Th2 cells induce humoral immunity by secreting IL-4, IL-5, IL-6, and IL-13 (Barnes, 2001). IL-4 and IL-5 are involved in activating inflammation in response to antigen exposure and are essential for activation, expansion, and differentiation of eosinophils (Chakir et al., 2003). IL-6 is recognized as a typical marker of inflammatory state (Rincon and Irvin, 2012). IL-13 is a central effect of Th2 responses and is necessary and sufficient to induce all the cardinal features of allergic lung inflammation and experimental asthma (Wills-Karp, 2000). It is widely acknowledged that Th2 cells are very important in the pathogenesis of asthma (Kim et al., 2010). Much research has indicated that asthma is characterized by chronic airway inflammation, which was driven by Th2 cytokines (Galli et al., 2008; Holgate, 2008). On the other hand, Th17 cells might be associated with asthma, and there has been an increasing interest in the role of Th17-dependent pathways in asthma (Nelson et al., 2003; Gibson and Foster, 2019). IL-17 is a cytokine produced by T lymphocytes and eosinophils within asthmatic airways. Peribronchiolar or subbasement membrane fibrosis is one of the several key features of airway remodeling in asthma (Molet et al., 2001). It has been suggested that IL-17 promotes the formation of subepithelial fibrosis and active fibro-blasts and macrophages for the secretion of TNF-α, IL-1β, and IL-6 (Lindén, 2001; Krishnamoorthy et al., 2018).

Network pharmacology is an emerging discipline based on the theory of systems biology, and it may exert a meaningful tool to characterize the target profiles and pharmacological mechanisms of TCM in detail (Jiao et al., 2021; Zhang et al., 2021). Molecular docking could determine the binding energy between the ligands and macromolecules (Xu et al., 2021). In this study, a combined bioinformatics analysis was applied to identify the core ingredient–target interactions and screen key pathways in asthma treatment of the PEF. A total of 21 active ingredients were filtered by ADME screening, among which are lignans and triterpenes. All of these compounds complied with Lipinski’s rule of five and possessed high gastrointestinal absorption, demonstrating that these compounds have good pharmacokinetic profiles. Furthermore, 129 overlapping targets of these compounds against asthma were obtained based on the PPI analysis. The top five targets were obtained with a high ranking of connectivity, namely, MAPK14, JUN, STAT3, MAPK3, and MAPK1. Among these cytokines, MAPK14, MAPK1, and MAPK3 are involved in a wide variety of cellular processes, such as proliferation, differentiation, transcription regulation, and development, and they all play pivotal roles in the regulation of proinflammatory cytokines (Di Paola et al., 2009; Yang et al., 2020). MAPK14, also called P38-α, is the prototype member of the P38 MAPK family. It plays an essential role in the cellular cascade reactions produced by proinflammatory cytokines or extracellular stimuli. Inflammatory cytokines, such as IL-6 and TNF-α, can regulate extracellular signal-regulated kinase (ERK) and NF-κB pathways by activating the P38 MAPK pathway and cascading to amplify the inflammatory response (Madkour et al., 2021). STAT3 is induced and phosphorylated by IL-6 or IL-10 cytokine signaling, and it plays a significant role in activation of CD4^+^ T cells and regulatory T cell populations (Hausding et al., 2011). STAT3 is essential for Th2 cytokine production and Th17 lymphocyte development, and STAT3 inhibition prevents airway inflammation by blocking the accumulations of Th2 and Th17 cells in a murine asthma model (Gavino et al., 2016).

Furthermore, the mechanism of action of PEF against asthma involving various targets and multiple biological processes was analyzed by the GO enrichment analysis and the KEGG pathways analysis. Our findings showed that the PEF can directly treat asthma through the IL-17 signaling pathway, Th17 cell differentiation, and the calcium signaling pathway. In this study, the IL-17 signaling pathway was found by the KEGG enrichment analysis to be an important pathway by which the PEF counteracted asthma, and 15 targets are involved (including the core targets MAPK1, MAPK14, MAPK3, and JUN). IL-17 is considered to be one of the significant cytokines of the Th17 pathway and is involved in neutrophilic inflammation and airway remodeling in severe asthma (Al-Ramli et al., 2009). Th17 cells can recruit neutrophil granulocytes in the lungs, and the activation could be by C-X-C Motif Chemokine Ligand 8 (CXCL8) production directly or through the production of IL-6, granulocyte-macrophage colony-stimulating factor (GM-CSF), CXCL8 by airway epithelial cells indirectly (Pelletier et al., 2010). Our study has found that there are 17 targets involved in the Th17 cell differentiation, and in this pathway, five core targets, MAPK14, JUN, STAT3, MAPK3, and MAPK1, are involved. In this work, the calcium signaling pathway was predicted to be one of the most enriched pathways with 21 targets involved. The calcium pathway modulates T cell differentiation and proinflammatory cytokines, and it plays vital a role in regulation of airway smooth muscle (Jude et al., 2008). Airway remodeling and airway hyperreactivity are two major characteristics of asthma. In an asthma environment, the airway smooth muscle (ASM) is hypercontractile. The intracellular calcium concentration is crucial for initiating and sustaining ASM contraction and is highly implicated in physiological pathways (Lee and Jeong, 2020). Research has shown that the expression and activity of the calcium pathway are regulated by proinflammatory cytokines, thereby contributing to hypercontractile ASM (Berair et al., 2013).

Molecular docking has become an important tool in computer-aided drug design over the last few years. Here, we used molecular docking to verify the interaction affinities between the top five targets (MAPK14, JUN, STAT3, MAPK3, and MAPK1) and related compounds. Most compounds demonstrated good affinities toward the five targets, which validated the network pharmacology results. It is noteworthy that nowadays, there have been few studies on the treatment of asthma with lignans and triterpenes. More attention might be paid to these compounds in asthma treatment in future studies.

In this study, combined approaches were applied to clear the pharmacodynamic substances and explain the anti-asthmatic effects of SSF. More comprehensive experimental validation and clinical studies are required to verify these results in the future. In addition, further optimization of structures and dosage forms may be required to increase the anti-asthmatic activity and oral bioavailability for its clinical applicability. Taken together, our findings might provide a pharmacodynamic substance basis and potential mechanisms for the use of lignans and triterpenes of SSF for asthma treatment.

## 5 Conclusion

In conclusion, this study may be the first to systematically study the pharmacodynamic material basis and potential mechanisms of the anti-asthmatic fraction of Schisandrae Sphenantherae Fructus. Our results have shown that the most potent anti-asthmatic fraction of SSF is the PEF. Subsequently, we found that the 21 significant active compounds identified from the PEF may attenuate the pathological and inflammatory changes in asthma through 129 overlapping targets and 10 pathways. Furthermore, MAPK14, JUN, STAT3, MAPK3, and MAPK1 might play important roles during anti-asthmatic progress, and the PEF might reduce inflammation by modulating Th2 cells, IL-17 signaling pathway, Th17 cell differentiation, and the calcium signaling pathway. This study highlights new insights into discovering active compounds with specific anti-asthmatic activity and provides a scientific basis for further experimental studies.

## Data Availability

The datasets presented in this study can be found in online repositories. The names of the repository/repositories and accession number(s) can be found in the article/Supplementary Material.
