# Supplementary material for: Anti-asthmatic fraction screening and mechanisms prediction of Schisandrae Sphenantherae Fructus based on a combined approach
Source: Front Pharmacol. 2022 Sep 12;13:902324. doi: 10.3389/fphar.2022.902324 (PMC9511055; doi:10.3389/fphar.2022.902324)
Supplement: Supplementary file 6 [file DataSheet2.docx]

Anti-asthmatic Fraction Screening and Mechanisms Prediction of Schisandrae Sphenantherae Fructus based on Combined Approach

**Fan Li^1^, Bin Li^1,2^, Jiushi Liu^1,2^, Xueping Wei^1,2^, Tingyan Qiang^1^, Xinlu Mu^1^, Yumeng Wang^1,3^, Yaodong Qi^1,2^, Bengang Zhang^1,2^, Haitao Liu^1,2^*, Peigen Xiao^1,2^**

**Supplementary Information**

**Materials and Methods (supplementary)**

**Materials and Reagents**

Powder of dried SSF was refluxed with 95%, 80%, and 65% ethanol (6L, 2h × 2). After filtration, the filtrates were concentrated under decompression and merged. Then the mixture was extracted with petroleum ether and ethyl acetate at room temperature respectively and was freeze-dried to obtain three SSF fractions, including PEF, EAC, and HPF.

Ethanol, petroleum ether, and acetonitrile (analytical grade reagents) were purchased from Beijing Chemical Corporation (Beijing, China). Methanol and acetonitrile (MS-grade) were purchased from Merck (Darmstadt, Germany). Pure water (18.2 MΩ·cm) for UPLC analysis was generated with a Milli-Q water purification system (Millipore, Bedford, MA, USA). Standards schisantherin E, gomisin J, schisandrin, pregomisin, Schisandrol B, gomisin G, schisantherin A, schisantherin B, schisanhenol, interiotherin A, deoxyschizandrin, d-epigalbacine, benzoylgomisin O, and angeloylgomisin H (unpublished work). Ovalbumin (OVA, grade V, A5503) and dexamethasone were purchased from Sigma-Aldrich (MO, USA). Imject™ Alum Adjuvant was purchased from Thermo Scientific (Waltham, MA, USA). The enzyme-linked immunosorbent assay (Elisa) kits of immunoglobulin E (IgE), interleukin (IL)-4, IL-5, IL-6, IL-13, IL-17 (Meimian, Jiangsu, China) were purchased from Beijing Yikebaide Technology Co., Ltd. (Beijing, China).

### UPLC-Q/TOF-MS/MS Methods

### Sample and Standard Preparation

SSF, PEF, EAC, and HPF were dissolved into 1 mL methanol and centrifuged at 14,000 rpm for 10 min. The supernatant was collected and filtered using a syringe filter (0.22 μm). Standard solutions (1 μg/mL) were prepared by dilution of stock solutions of each reference substance (0.1 mg/mL in methanol).

**In-House Database Construction**

A three-phase approach was applied to construct an in-house database of compounds from *Schisandra sphenanthera*. Firstly, the compounds previously reported from *Schisandra sphenanthera* were retrieved through multiple databases, including SciFinder, PubMed, Web of Science, Google scholar, and CNKI (accessed on 1 December 2021-10 December 2021). Then, 2D structures (.mol) of these compounds were obtained by searching the name or structure in SciFinder. Finally, all files (.mol) were integrated into an in-house database with Progenesis SDF Studio (Supplementary Table 1).

UPLC chromatography Analysis

**Sample and Standard Preparation, and Chromatographic Separation**

The methods of sample and standard preparation, and chromatographic separation were the same as sections “Sample and Standard Preparation” and 2.2.1.

**Network Pharmacology Analysis**

**Asthma-Related Targets Collection**

The settings of GEO database were kept with the following specifications: The "Expression profiling by array" was considered as the screening standard, and the criteria of differentially expressed genes (DEGs) were as follows: Adjusted *P* < 0.05 and | Log (Fold Change) |>1.3 (Barrett, 2004). The raw data were downloaded as MINiML files. The differentially expressed mRNA was then studied by the R software limma package, and the ggplot2 and pheatmap packages were used to draw volcano plots and heatmap of DEGs.

**Protein-Protein Interaction (PPI) analysis**

The settings of STRING database were kept with the following specifications: The organism was set to “Homo sapiens”, and the minimum required interaction score > 0.9 was chosen as significant. The thickness of an edge represents the combined score, and the degree represents the number of other nodes connected directly to a node. The higher the degree is, the more important the node is.

### Enrichment Analysis, Inference of Upstream Pathway Activity, and Network Construction

The settings of webgestalt database were kept with the following specifications: “Homo sapiens” was chosen as the organism of interest, over-representation analysis (ORA) was set as the method of interest, and “agilent wholegenome” was selected as the reference set.

The settings of SPEED2 online tool were kept with the following specifications: The “bates test” was selected as test statistics for enrichment. The colors indicated the adjusted *P*-value, and the ranked lists about activity were determined by the absolute *P*-value, the bright the color, the higher the ranking.
