# Supplementary material for: Anti-asthmatic fraction screening and mechanisms prediction of Schisandrae Sphenantherae Fructus based on a combined approach
Source: Front Pharmacol. 2022 Sep 12;13:902324. doi: 10.3389/fphar.2022.902324 (PMC9511055; doi:10.3389/fphar.2022.902324)
Supplement: Supplementary file 8 [file DataSheet1.docx]

Supplementary Material


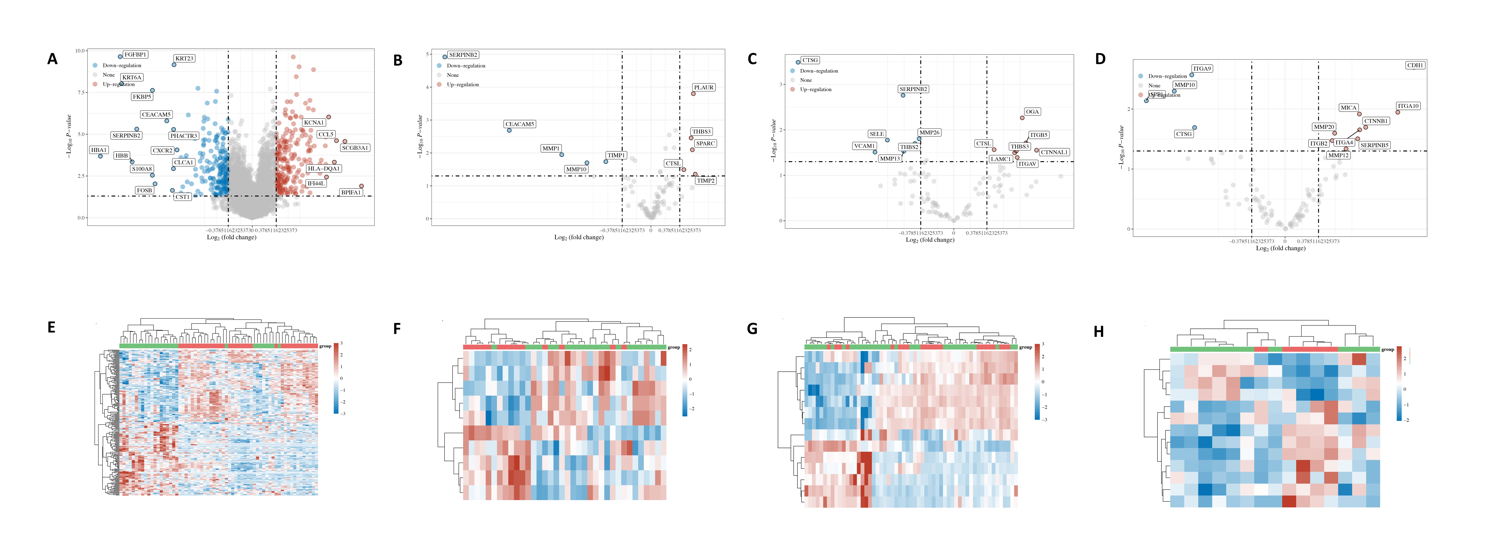


**Supplementary Figure 1.** Volcano plots and hierarchical clustering analysis of differentially expressed genes (DGEs) from the GEO database. (A) and (E) from GSE64913, (B) and (F) from GSE67472, (C) and (G) from GSE104468, (D) and (H) from GSE27876. The red dots represent up-regulated genes, blue represent down-regulated.

| **Supplementary Table 1. Compounds of In-House Database of Schisandra sphenanthera.** | | | | |  |  |  |
| --- | --- | --- | --- | --- | --- | --- | --- |
| **No.** | **Compounds** | **Classification** | **No.** | **Compounds** | | **Classification** | |
| 1 | schisantherin A | dibenzocyclooctadienes | 90 | *meso*-dihydroguaiaretic acid | | dibenzylbutanes | |
| 2 | schisantherin B | dibenzocyclooctadienes | 91 | (7,8-*trans*-8,8'-*cis*)-7-(3,4-dihydroxyphenyl)-7'-(3,4-methylenedioxyphenyl)-8, 8'-dimethyl- butan-7-ol | | dibenzylbutanes | |
| 3 | benzoylgomisin Q | dibenzocyclooctadienes | 92 | myristargenol A | | dibenzylbutanes | |
| 4 | schisantherin C | dibenzocyclooctadienes | 93 | schisphenlignan F | | dibenzylbutanes | |
| 5 | schisantherin D | dibenzocyclooctadienes | 94 | (-)-secoisolariciresinol-O-a-L-rhamnopyranoside | | dibenzylbutanes | |
| 6 | schisantherin E | dibenzocyclooctadienes | 95 | (-)-secoisolariciresinol 9-O-α-L-arabinopyranoside | | dibenzylbutanes | |
| 7 | gomisin F | dibenzocyclooctadienes | 96 | Matairesinol 4'-O-glucoside | | dibenzylbutanes | |
| 8 | schirubrisin B | dibenzocyclooctadienes | 97 | schisandrone | | aryltetralins | |
| 9 | schisanwilsonin G | dibenzocyclooctadienes | 98 | schisphentetralone A | | aryltetralins | |
| 10 | schisanwilsonin D | dibenzocyclooctadienes | 99 | (-)-epiaristoligone | | aryltetralins | |
| 11 | schisanwilsonin B | dibenzocyclooctadienes | 100 | 3',4'-dimethoxybenzoic acid-(3'',4''-dimethoxyphenyl)-2-methyl -3-oxobutylester | | 7, 8-seco lignans | |
| 12 | schispenthin E | dibenzocyclooctadienes | 101 | schisandlignan A | | 7, 9-seco lignans | |
| 13 | gomisin P | dibenzocyclooctadienes | 102 | schisandlignan B | | 7, 10-seco lignans | |
| 14 | gomisin Q | dibenzocyclooctadienes | 103 | schisandlignan C | | 7, 11-seco lignans | |
| 15 | gomisin G | dibenzocyclooctadienes | 104 | schisphenone | | other lignans | |
| 16 | sphaerandrin A | dibenzocyclooctadienes | 105 | schisphenlignan E | | other lignans | |
| 17 | gomisin B | dibenzocyclooctadienes | 106 | anwuweizic acid | | lanostanes | |
| 18 | benzoylgomisin U | dibenzocyclooctadienes | 107 | schisanol | | lanostanes | |
| 19 | tigloylgomisin P | dibenzocyclooctadienes | 108 | schisanlactone G | | lanostanes | |
| 20 | angeloylgomisin P | dibenzocyclooctadienes | 109 | schisanlactone H | | lanostanes | |
| 21 | schisphenthin D | dibenzocyclooctadienes | 110 | manwuweizic acid | | lanostanes | |
| 22 | schisphenlignan A | dibenzocyclooctadienes | 111 | schisanlactone C | | lanostanes | |
| 23 | schisphenlignan B | dibenzocyclooctadienes | 112 | schisanlactone D | | lanostanes | |
| 24 | schisphenlignan C | dibenzocyclooctadienes | 113 | kadsuric acid 3-methylester | | lanostanes | |
| 25 | schisphenlignan L | dibenzocyclooctadienes | 114 | schisphenthin A | | lanostanes | |
| 26 | schisphenin C | dibenzocyclooctadienes | 115 | schisphenthin B | | lanostanes | |
| 27 | schisphenin D | dibenzocyclooctadienes | 116 | schisphenthin C | | lanostanes | |
| 28 | schisphenlignan D | dibenzocyclooctadienes | 117 | kadsuric acid | | lanostanes | |
| 29 | schisphenlignan M | dibenzocyclooctadienes | 118 | coccinic acid | | lanostanes | |
| 30 | schisphenlignan N | dibenzocyclooctadienes | 119 | sphenasin A | | cycloartanes | |
| 31 | chloromethyl schisantherin B | dibenzocyclooctadienes | 120 | micranoic acid B | | cycloartanes | |
| 32 | schisandlignan D | dibenzocyclooctadienes | 121 | isoschizandronic acid | | cycloartanes | |
| 33 | methylgomisin O | dibenzocyclooctadienes | 122 | schizandronic acid | | cycloartanes | |
| 34 | methylgomisin R | dibenzocyclooctadienes | 123 | wuweizilactone acid A | | cycloartanes | |
| 35 | gomisin U | dibenzocyclooctadienes | 124 | schinalactone A | | cycloartanes | |
| 36 | benzoylgomisin U | dibenzocyclooctadienes | 125 | schinalactone B | | cycloartanes | |
| 37 | tigloylgomisin O | dibenzocyclooctadienes | 126 | schinalactone C | | cycloartanes | |
| 38 | epigomisin O | dibenzocyclooctadienes | 127 | schisphendilactone A | | cycloartanes | |
| 39 | schisphenin E | dibenzocyclooctadienes | 128 | schisphendilactone B | | cycloartanes | |
| 40 | isogomisin O | dibenzocyclooctadienes | 129 | nigranoic acid | | cycloartanes | |
| 41 | schisanwilsonin O | dibenzocyclooctadienes | 130 | schisanlactone B | | cycloartanes | |
| 42 | schisandrol B | dibenzocyclooctadienes | 131 | schisanlactone A | | cycloartanes | |
| 43 | 6-O-benzoylgomisin O | dibenzocyclooctadienes | 132 | kadsulactone A | | cycloartanes | |
| 44 | angeloylgomisin H | dibenzocyclooctadienes | 133 | propinic lactone A | | cycloartanes | |
| 45 | angeloylgomisin O | dibenzocyclooctadienes | 134 | lancifoic acid A | | cycloartanes | |
| 46 | benzoylisogomisin Q | dibenzocyclooctadienes | 135 | pre-schisanartanin E | | nortriterpenes | |
| 47 | interiotherin A | dibenzocyclooctadienes | 136 | pre-schisanartanin F | | nortriterpenes | |
| 48 | isoschizandrin | dibenzocyclooctadienes | 137 | pre-schisanartanin G | | nortriterpenes | |
| 49 | schizandrin | dibenzocyclooctadienes | 138 | pre-schisanartanin H | | nortriterpenes | |
| 50 | angeloylgomisin Q | dibenzocyclooctadienes | 139 | pre-schisanartanin I | | nortriterpenes | |
| 51 | interiotherin B | dibenzocyclooctadienes | 140 | pre-schisanartanin J | | nortriterpenes | |
| 52 | schisphenin F | dibenzocyclooctadienes | 141 | sphenadilactone A | | nortriterpenes | |
| 53 | schisandrin B | dibenzocyclooctadienes | 142 | sphenadilactone B | | nortriterpenes | |
| 54 | rubrisandrin A(1a) | dibenzocyclooctadienes | 143 | sphenadilactone C | | nortriterpenes | |
| 55 | rubrisandrin A (1b) | dibenzocyclooctadienes | 144 | sphenadilactone D | | nortriterpenes | |
| 56 | gomisin J | dibenzocyclooctadienes | 145 | sphenadilactone E | | nortriterpenes | |
| 57 | (+)-gomisin K_3_ | dibenzocyclooctadienes | 146 | sphenadilactone F | | nortriterpenes | |
| 58 | gomisin M_2_ | dibenzocyclooctadienes | 147 | sphenalactone A | | nortriterpenes | |
| 59 | schisandrin A | dibenzocyclooctadienes | 148 | sphenalactone B | | nortriterpenes | |
| 60 | schisanhenol | dibenzocyclooctadienes | 149 | sphenalactone C | | nortriterpenes | |
| 61 | wuweizisu C | dibenzocyclooctadienes | 150 | sphenalactone D | | nortriterpenes | |
| 62 | chicanine | tetrahydrofurans | 151 | schintrilactone C | | nortriterpenes | |
| 63 | d-epigalbacine | tetrahydrofurans | 152 | schintrilactone D | | nortriterpenes | |
| 64 | ganschisandrine | tetrahydrofurans | 153 | schirubridilactone A | | nortriterpenes | |
| 65 | (-)-machilusin | tetrahydrofurans | 154 | schirubridilactone D | | nortriterpenes | |
| 66 | (7*S*,8*S*,7'*R*,8'*S*)-7-(3,4-methylenedioxy-phenyl)-8-methyl-8'-methyl-7'-(3',4'- dihydroxy-phenyl)-tetrahydrofuran | tetrahydrofurans | 155 | propindilactone A | | nortriterpenes | |
| 67 | (-)-talaumidin | tetrahydrofurans | 156 | propindilactone D | | nortriterpenes | |
| 68 | veraguensin | tetrahydrofurans | 157 | micrandilactone D | | nortriterpenes | |
| 69 | schisphenlignan G | tetrahydrofurans | 158 | micrandilactone E | | nortriterpenes | |
| 70 | schiglaucin B | tetrahydrofurans | 159 | lancifodilactone B | | nortriterpenes | |
| 71 | schiglaucin A | tetrahydrofurans | 160 | lancifodilactone C | | nortriterpenes | |
| 72 | epoxyzuonin A | tetrahydrofurans | 161 | lancifodilactone O | | nortriterpenes | |
| 73 | schisphenlignan H | tetrahydrofurans | 162 | lancifodilactone N | | nortriterpenes | |
| 74 | henricine A | tetrahydrofurans | 163 | lancifodilactone G | | nortriterpenes | |
| 75 | (7*S*,8*S*,7'*R*,8'*S*)-7-methoxyl-7-(3,4-methyl-enedioxyphenyl)-8-hydroxyl-7'-(5'-hydroxy-3'-methoxyphenyl)-8'-methyl-tetrahydrofuran | tetrahydrofurans | 164 | henridilactone A | | nortriterpenes | |
| 76 | (7*S*,8*S*,7'*R*,8'*S*)-7-methoxyl-7-(3,4-methyl-enedioxy-phenyl)-8-hydroxyl-8'-methyl-7'-(3',4',5'-trimethoxyphenyl)-tetrahydrofuran | tetrahydrofurans | 165 | kadsuphilactone A | | nortriterpenes | |
| 77 | (7*S*,8*S*,7'*R*,8'*S*)-7-methoxyl-7-(3,4-methyl-enedioxyphenyl)-8-hydroxyl-8'-methyl-7'-(3',4'-methylenedioxyphenyl)-tetrahydrofuran | tetrahydrofurans | 166 | cuparene | | volatile oils | |
| 78 | (7*S*,8*S*,7'*R*,8'*S*)-(7,8-trans-8,8'-trans-7',8'-trans)-7-methoxyl-7-(3,4-methylenedioxy-phenyl)-8-methyl-8'-methyl-7'-(3',4'-dimethoxy-phenyl)-tetrahydrofuran | tetrahydrofurans | 167 | thujopsene | | volatile oils | |
| 79 | (7*S*,8*S*,7'*R*,8'*S*)-7-methoxyl-7-(3,4,5-trimethoxyphenyl)-8methyl-8'-methyl-7'-(3',4'-methylenedioxyphenyl)-tetrahydrofuran | tetrahydrofurans | 168 | α-santalene | | volatile oils | |
| 80 | (7*S*,8*S*,7'*R*,8'*S*)-7,8-epoxide-7-(3,4-methyl-enedioxyphenyl)-8-methyl-8'-methyl-7'-(3',4'-dimethoxyphenyl)-tetrahydrofuran | tetrahydrofurans | 169 | β-selinene | | volatile oils | |
| 81 | (7*S*,8*S*,7'*R*,8'*S*)-7,8-epoxide-7-(3,4-methylenedioxyphenyl)-8-methyl-8'-methyl-7'-(4'-hydroxy-3'-methoxyphenyl)-tetrahydrofuran | tetrahydrofurans | 170 | σ-elemene | | volatile oils | |
| 82 | schisphenlignan I | tetrahydrofurans | 171 | β-himachalen | | volatile oils | |
| 83 | schisphenlignan J | tetrahydrofurans | 172 | γ-cadinene | | volatile oils | |
| 84 | schisphenlignan K | tetrahydrofurans | 173 | ylangene | | volatile oils | |
| 85 | (+)-1-hydroxy pinoresinol | tetrahydrofurans | 174 | IH-benzocycloheptene | | volatile oils | |
| 86 | pregomisin | dibenzylbutanes | 175 | 2, 4a, 5, 6, 7, 8-hexahydro-3, 5, 5, 9-tetramethyl | | volatile oils | |
| 87 | (+)-anwulignan | dibenzylbutanes | 176 | ledene oxide-Ⅱ | | volatile oils | |
| 88 | dl-anwulignan | dibenzylbutanes | 177 | alloaromadendrene oxide-Ⅱ | | volatile oils | |
| 89 | sphenanlignan | dibenzylbutanes |  |  | |  |  |
